# Neuroprotective Effects of *Asparagus officinalis* Stem Extract in Transgenic Mice Overexpressing Amyloid Precursor Protein

**DOI:** 10.1155/2021/8121407

**Published:** 2021-05-10

**Authors:** Zhanglong Peng, Supinder Bedi, Vivek Mann, Alamelu Sundaresan, Kohei Homma, Gregory Gaskey, Minoru Kowada, Shahid Umar, Anil D. Kulkarni, Holger K. Eltzschig, Marie-Francoise Doursout

**Affiliations:** ^1^Department of Anesthesiology, McGovern Medical Houston, TX, USA; ^2^Pediatric Surgery, McGovern Medical Houston, TX, USA; ^3^Department of Biology, Texas Southern University, Houston, TX, USA; ^4^Amino Up, Sapporo, Japan; ^5^General Surgery, McGovern Medical Houston, TX, USA; ^6^Department of Surgery, University of Kansas, Kansas City, KS, USA

## Abstract

To mimic Alzheimer's disease, transgenic mice overexpressing the amyloid precursor protein (APP) were used in this study. We hypothesize that the neuroprotective effects of ETAS®50, a standardized extract of *Asparagus officinalis* stem produced by Amino Up Co., Ltd. (Sapporo, Japan), are linked to the inhibition of the apoptosis cascade through an enhancement of the stress-response proteins: heat shock proteins (HSPs). APP-overexpressing mice (double-transgenic APP and PS1 mouse strains with a 129s6 background), ages 6-8 weeks old, and weighing 20-24 grams were successfully bred in our laboratory. The animals were divided into 5 groups. APP-overexpressing mice and wild-type (WT) mice were pretreated with ETAS®50 powder (50% elemental ETAS and 50% destrin) at 200 mg/kg and 1000 mg/kg body weight. Saline, the vehicle for ETAS®50, was administered in APP-overexpressing mice and WT mice. ETAS®50 and saline were administered by gavage daily for 1 month. Cognitive assessments, using the Morris Water Maze, demonstrated that memory was recovered following ETAS®50 treatment as compared to nontreated APP mice. At euthanization, the brain was removed and HSPs, amyloid *β*, tau proteins, and caspase-3 were evaluated through immunofluorescence staining with the appropriate antibodies. Our data indicate that APP mice have cognitive impairment along with elevated amyloid *β*, tau proteins, and caspase-3. ETAS®50 restored cognitive function in these transgenic mice, increased both HSP70 and HSP27, and attenuated pathogenic level of amyloid *β*, tau proteins, and caspsase-3 leading to neuroprotection. Our results were confirmed with a significant increase in HSP70 gene expression in the hippocampus.

## 1. Introduction

Alzheimer's disease (AD) is a degenerative brain disease and the most common cause of dementia, affecting approximately 47 million people worldwide. This number is expected to increase to over 115 million by 2050 [1, 2]. The outcomes of this disease result in irreversible loss of cortical neurons, primarily in the associative cortex and hippocampus. It is characterized by a decline in memory, language, problem-solving, and other cognitive skills that affect a person's ability to perform everyday activities. Researchers have shown that many metabolic disorders and injuries including stroke, neurodegenerative disease, epilepsy, and shock induce a stress response that leads to the release of various proteins, particularly the 70 kD heat shock protein (HSP70) [3–6]. Because high levels of HSP70 expression were reported in the brain following injury, investigators postulated that HSP70 might offer some neuroprotective effects [3, 7, 8]. Son et al. [9] have reported that the expression of two HSPs, specifically HSP70 and HSP27, is prominent in the brain because both are highly induced in glial cells and neurons following various harmful stimuli in the early neurodegeneration stage. Experiments using animal stroke models and tissue culture systems have shown that the overexpression of HSP70 diminished ischemic injury and protected both neurons and glial cells [10–12]. Numerous studies have recognized the ability of HSPs in reducing apoptosis following various stimuli such as heat, DNA damage, and death receptor ligation [13, 14].

Ito and collaborators have recently developed a new functional material, *Asparagus officinalis extract* (ETAS®50), from unused parts of asparagus grown in Hokkaido, Japan [15]. In the proposed experiments, ETAS®50 (50% of elemental ETAS and 50% Dextrin) was studied as a whole compound [16]. Using senescence-accelerated mice, Sakurai et al. [17] have revealed that ETAS®50 showed neuroprotective effects and attenuated cognitive impairment. In a pilot and small-sized human study, Ito et al. [18] reported that in healthy volunteers consuming up to 150 mg/d of ETAS®50 daily for 7 days, mRNA expression of HSP70 in peripheral leukocytes was significantly elevated at intakes of 100 or 150 mg/d, compared to baseline levels. Since HSP70 is recognized to be a stress-linked protein and its induction leads to cytoprotection, the authors suggested that ETAS®50 exerted antistress effects under stressful conditions, resulting from an enhancement of HSP70 expression [15]. To reinforce their findings, Ito et al. conducted a toxicology assessment of ETAS®50 in rats [18]. The authors concluded that ETAS®50 did not cause genetically mutagenic effects in the bacterial reverse mutation test, nor in the *in vivo* micronucleus assay. Therefore, the study supported the safety of ETAS®50 as a food and dietary supplement.

As previously reported by Takeda et al. [19], the SAMP8 mice are a spontaneous animal model of overproduction of amyloid precursor protein (APP) and oxidative damage. Simón et al. [20] reported that transgenic mice expressing mutant human APP developed an age-dependent amyloid pathology and memory deficits, but no obvious neuronal loss. Because our current knowledge on the mechanisms of ETAS®50 and brain function are limited, transgenic mice overexpressing APP were used in our studies, as previously described by Simón et al. [20] while elucidating the mechanisms of Alzheimer disease [21].

Until now, no investigations have reported the relationship among HSPs, the apoptosis pathway, and stress conditions in the hippocampus. Whether the underlying mechanisms of ETAS®50 are related to HSP overproduction leading to apoptosis inhibition in APP-overexpressed mice remains unknown. It is now well established that the hippocampus is viewed as a major brain region for its involvement in stressful conditions, but also for the control of emotion and cognition [22].

The overall goal of this proposal was to assess the correlation between stress response proteins such as HSP levels and cognitive function in transgenic mice overexpressing APP fed with ETAS®50 over several weeks. Taken together, we postulated that ETAS®50 would support a possible neuroprotective role through HSP enhancement, restoring learning and global intellectual performance.

## 2. Experimental Design

### 2.1. Materials and Methods

All procedures were approved by the University of Texas Animal Welfare Committee (AWC-16-0050) and were consistent with the National Institutes of Health “Guide for the Care and Use of Laboratory Animals”. APP-overexpressing founder mice (5 pairs) were kindly provided by Dr. Claudio Soto, Ph.D., in the Department of Neurology at the University of Texas Health Science Center at Houston. Six to 8 weeks old, male and female, 20-24 grams APP-overexpressing mice (a double transgenic APP and PS1 mouse strain with 129s6 background) were used in the proposed studies. Specifically, mice have the Swedish mutation of APP and deltaE9 mutation in PS1. APP/PS1 are double transgenic mice expressing a chimeric mouse/human amyloid precursor protein (Mo/HuAPP695swe) and a mutant human presenilin 1 (PS1-dE9), both directed to CNS neurons. Both mutations are associated with early-onset Alzheimer's disease. As such, these mice may be useful in studying neurological disorders of the brain, specifically Alzheimer's disease, amyloid plaque formation, and aging [23, 24]. Mice were bred and acclimatized in our animal facility with an ambient temperature (22 ± 2°C) per the guidelines for “Care and Use of Animals in Scientific Research.” ETAS®50 was manufactured according to a method previously described.

**Figure 1:**
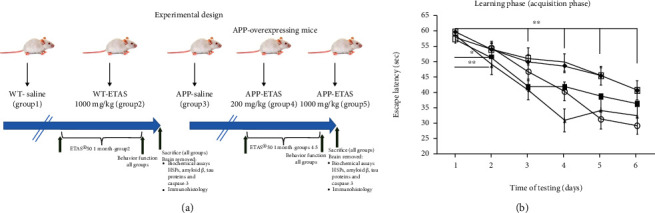
(a) Experimental design—scheme of animal groups's description; (b) effects of saline and/or ETAS (200 mg/kg and 1000 mg/kg) on APP-overexpressed mice and WT mice (1000 mg/kg) on escape latency (acquisition phase) using the Morris Water Maze (*n* = 10 mice in each group). ^∗∗^*p* < 0.01 APP saline, APP-ETAS 200 and 1000 mg/kg; WT saline and WT-ETAS 1000 mg/kg at days 3–6 vs. day 1; ^∗∗^*p* < 0.01 APP-ETAS 1000 mg/kg at day 2 vs. day 1; ^∗^*p* < 0.05 APP-ETAS 200 mg/kg day 2 vs. day 1. ♦ APP saline; ■ APP-ETAS 200 mg/kg; ▲ APP-ETAS 1000 mg/kg; □ WT saline and ○WT-ETAS 1000 mg/kg; Sec: seconds.

To study the effects of ETAS®50 on cognitive function and stress hormones, animals were divided into 5 groups. Ten (10) animals were included in each group for a total of 50 mice. Group 1 wild-type (WT) mice received saline; group 2 WT mice received ETAS®50 only at a higher dose of 1000 mg/kg body weight; group 3 APP-overexpressing mice received saline; group 4 APP-overexpressing mice received ETAS®50 at a low dose of 200 mg/kg body weight, whereas group 5 APP-overexpressing mice received ETAS®50 at a higher dose of 1000 mg/kg body weight. Both ETAS®50 and saline were administered by gavage daily for 1 month (Figure 1(a)).

### 2.2. Cognitive Assessment—Learning and Memory Tests

Following ETAS®50 feeding, mice were subjected to a cognitive assessment using the Morris Water Maze (MWM). Briefly, the MWM was described 20 years ago as a device to investigate spatial learning and memory in laboratory rats [25]. The MWM is one of the most widely used tasks in behavioral neuroscience for studying the psychological processes and neural mechanisms of spatial learning and memory. Briefly, mice were gently introduced to a circular pool and were required to escape from water onto a hidden platform. The location of the platform can be identified only by using spatial memory. The mouse swam around the pool until it found the platform to stand on. Various shapes were used as external clues around the pool, displayed at the same place to help the mouse learn where the platform was located. The investigators measured how long it took for a mouse to find the hidden platform. This test was used at 1 month following ETAS®50 or saline feeding. Specifically, we used 4 start sites while performing the MWM test. Mice were given a series of daily trials using a semirandom set of start sites as represented in Table 1. Mice remained one minute at each site. The learning phase or (acquisition phase) lasted over 6 days. The retention phase or (probe phase) occurred on day 7. Only one site was used, and the duration of the trial lasted 1 minute. Ten mice were used for each trial.

Following the MWM test, animals from all groups were euthanized.

### 2.3. Immunofluorescence Analysis HSP70 and HSP27: Amyloid Beta (A*β*), Tau Protein, and Caspase-3

Mice were anesthetized with isoflurane (5%), and brains were removed, frozen in liquid nitrogen, then embedded in optimal cutting temperature (OCT), and stored at -80°C. Brain sections of 5 *μ*m were cut and mounted on super frost plus slides. Immunofluorescence staining proceeded as follows. The sections were air-dried at room temperature for 30 minutes, fixed in cold acetone at -20°C for 20 minutes, air-dried again, washed with phosphate-buffered saline (PBS), and blocked with blocking solution containing 5% normal bovine serum at room temperature for 30 min. Then, sections were, respectively, incubated with primary antibodies overnight at 4°C, including HSP70 monoclonal antibody (1 : 100; MA3-006, Thermo Fisher Scientific, Rockford, IL, USA), HSP27 polyclonal antibody (1 : 100; PA1-018, Thermo Fisher Scientific, Rockford, IL, USA), tau protein monoclonal antibody (1 : 100, RH233996, Thermo Fisher Scientific, Rockford, IL, USA), amyloid beta (A*β*) polyclonal antibody (2 *μ*/ml, SA243371, Thermo Fisher Scientific, Rockford, IL, USA), and caspase-3 monoclonal antibody (2 *μ*g/ml,SF256052, Thermo Fisher Scientific, Rockford, IL, USA). Subsequent to rinsing with PBS, sections were incubated with secondary antibodies (1 : 500, A32731, goat anti-Rabbit IgG Alexa Fluor Plus 488, Invitrogen, Rockford, IL, USA; 1 : 500, A32723, goat anti-mouse IgG Rockford, IL, USA) for 1 h at room temperature. After three further washes in PBS, the sections were mounted with Prolong gold antifade reagent with DAPI (P36935, Invitrogen, Rockford, IL, USA). Four mice per group and four sections per mouse were used to perform the immunofluorescence staining using a fluorescent microscope (Nikon Eclipse Ti, Melville, NY, USA) at ×200. Quantification was achieved using the Image J software (NIH).

To confirm the effects of *β*-amyloid, an ELISA quantification was performed. Specifically, the brain was homogenized and the ELISA analysis (soluble and insoluble *β*-amyloid 42) was performed following the kit instruction (KHB344, Invitrogen, Thermo Fisher Scientific, Waltham, MA, USA).

### 2.4. RNA Extraction and Purification: Gene Expression Quantification Using the Agilent Gene Array

Total RNA was extracted from the hippocampus of all groups of mice (*n* = 10 mice per group) with the use of RNeasy microarray tissue mini kits (Qiagen, Germantown, MD) and cleaned by TURBO DNase (Life Technologies/Thermo Fisher Scientific, Grand Island, NY). The purity of RNA (OD260/280 and OD260/230) was measured by NanoDrop 2000c spectrophotometer (Thermo Fisher Scientific, Waltham, MA). RNA integrity was evaluated on a Bioanalyzer 2100 (Agilent Technologies, Santa Clara, CA). RNA integrity number (RIN) values below 6 were considered failed and not sent for microarray processing. One hundred fifty nanograms of total RNA was converted to cDNA, amplified and labelled using the low input quick amp one-color labelling kits from Agilent following the manufactures protocol. A total of 1.65 *μ*g of labelled cDNA was hybridized to Agilent single-color mouse (V2) gene expression 4 × 44 K slides (Agilent Technologies). The arrays were preprocessed, and captured image data for microarray features was accomplished with Agilent's SureScan and Feature Extraction software. Data was background corrected with the local background algorithms in the Feature Extractor software. The main software used for subsequent analysis was BRB Array tools (v 4.1). Data was normalized using qsSuantile normalization, and transcripts that were differentially expressed (minimum of 1.2-fold across groups), identified by univariate analysis at *p* < 0.001, were corrected for multiple testing (Bonferroni). Transcripts that were identified as significantly different by this process were further analyzed by Ingenuity Pathway Analysis (Qiagen BioInformatics, Redwood City, CA, USA).

### 2.5. Statistical Analysis

#### 2.5.1. *In Vivo* Data

Data was analyzed by a one-way analysis of variance (ANOVA) to assess overall significance among groups. When differences were significant, multiple pairwise-comparisons were performed using Dunnett's *t*-test. The target sample size of 10 mice in each group was chosen to provide a 90% power analysis and to detect the size of the effect at 1.33 between control and treatments. Data was expressed as mean ± standard of the mean (SEM). ^∗^*p* < 0.05 was considered significant.

#### 2.5.2. *In Vitro* Data

Data was background corrected with the local background algorithms in the Feature Extractor software. The main software used for subsequent analysis was BRB Array tools (v 4.1). Data was normalized using quantile normalization, and transcripts that were differentially expressed (minimum of 1.2-fold across groups) were identified by univariate analysis at ^∗∗^*p* < 0.01, corrected for multiple testing (Bonferroni).

## 3. Results

**Figure 2:**
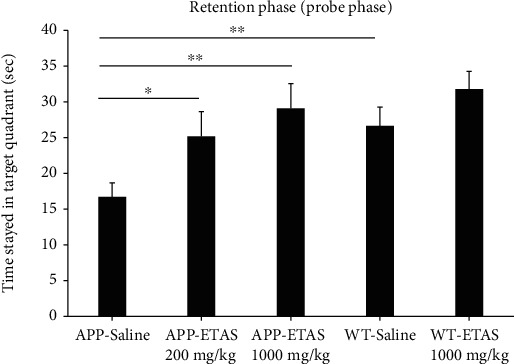
Representation of time stayed in target quadrant (probe phase), 7 days after removing the platform in APP-overexpressed mice treated with ETAS (200 mg/kg and 1000 mg/kg), respectively, and WT mice treated with ETAS (1000 mg/kg) (*n* = 10 mice in each group). ^∗^*p* < 0.05 APP-ETAS (200 mg/kg) vs. APP saline-treated mice; ^∗∗^*p* < 0.01 APP-ETAS (1000 mg/kg) vs. APP saline-treated mice; ^∗∗^*p* < 0.01 WT saline vs. APP saline-treated mice; Sec: seconds.

**Figure 3:**
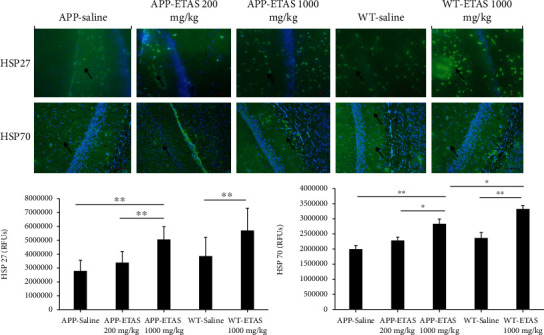
Quantification of heat shock protein (HSP27) and HSP70 in the hippocampus of APP-overexpressed mice treated with ETAS (200 mg/kg and 1000 mg/kg) and WT mice treated with ETAS (1000 mg/kg) for 1 month, respectively; RFUs: relative fluorescence units; green represents HSP27/70 expression, and blue represents DAPI. Arrows represent fluorescent green staining, indicating positive expression. Magnification is represented ×200. HSP27: ^∗∗^*p* < 0.01 APP-ETAS (1000 mg/kg) vs. APP-ETAS (200 mg/kg) and APP saline-treated mice; ^∗∗^*p* < 0.01 WT-ETAS (1000 mg/kg) vs. WT saline-treated mice; RFUs: relative fluorescence units. Four mice per group and four sections per mouse were used. HSP70: ^∗^*p* < 0.05 APP-ETAS (1000 mg/kg) vs. APP-ETAS (200 mg/kg); ^∗∗^*p* < 0.01 APP-ETAS (1000 mg/kg) vs. APP saline-treated mice; ^∗∗^*p* < 0.01 WT-ETAS (1000 mg/kg) vs. WT saline-treated mice. RFUs: relative fluorescence units. Four mice per group and four sections per mouse were used.

**Figure 4:**
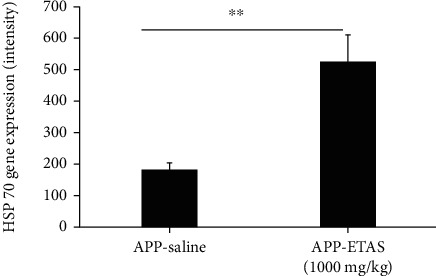
Representation of heat shock protein 70 (HSP70) gene expression (intensity units) in APP mice treated with ETAS (1000 mg/kg) vs. APP saline-treated mice. ^∗∗^*p* < 0.01 APP+ETAS (1000 mg/kg) vs. APP saline-treated mice. Samples were run in duplicate; *N* = 10 mice in each group.

**Figure 5:**
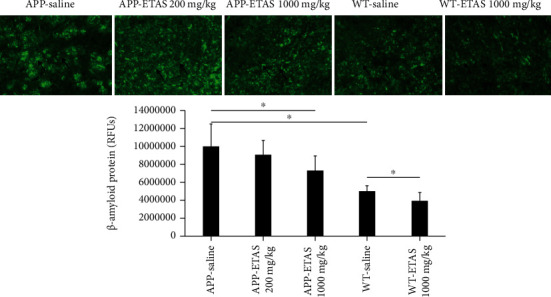
Quantification of *β*-amyloid protein in the hippocampus of APP-overexpressed mice treated with ETAS (200 mg/kg and 1000 mg/kg) and WT mice treated with ETAS (1000 mg/kg) for 1 month, respectively. ^∗^*p* < 0.05 APP-ETAS (1000 mg/kg) vs. APP saline-treated mice ^∗^*p* < 0.05 WT-ETAS (1000 mg/kg) vs. WT saline-treated mice; RFUs: relative fluorescence units. Four mice per group and four sections per mouse were used; green represents *β*-amyloid protein, and blue represents DAPI. Arrows represent fluorescent green staining, indicating positive expression. Magnification is represented ×200.

**Figure 6:**
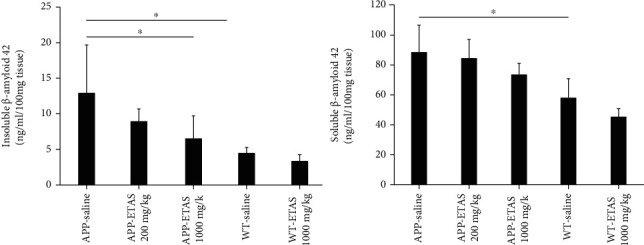
Quantification of soluble and insoluble *β*-amyloid using ELISA assay in the hippocampus in APP-overexpressed mice treated with ETAS (200 mg/kg and 1000 mg/kg) and WT mice treated with ETAS (1000 mg/kg) for 1 month, respectively. Insoluble: ^∗^*p* < 0.05 WT saline vs. APP saline; APP-ETAS 200 mg/kg vs. APP saline. Soluble: ^∗^*p* < 0.05 WT saline vs. APP saline (*n* = 5 mice in each group).

**Figure 7:**
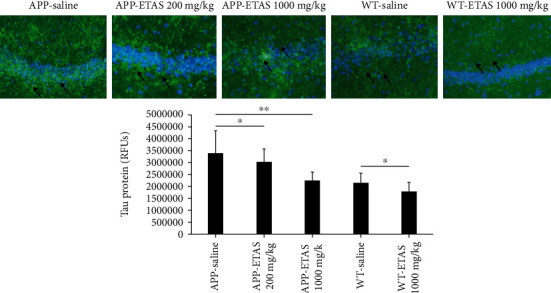
Quantification of tau protein in the hippocampus of APP-overexpressed mice treated with ETAS (200 mg/kg and 1000 mg/kg) and WT mice treated with ETAS (1000 mg/kg) for 1 month, respectively. ^∗^*p* < 0.05 APP-ETAS (200 mg/kg) vs. APP saline-treated mice; ^∗∗^*p* < 0.01 APP-ETAS (1000 mg/kg) vs. APP saline; ^∗^*p* < 0.05 WT-ETAS (1000 mg/kg) vs. WT saline-treated mice; RFUs: relative fluorescence units. Four mice per group and four sections per mouse were used. Green represents tau protein, and blue represents DAPI. Arrows represent fluorescent green staining, indicating positive expression. Magnification is represented ×200.

**Figure 8:**
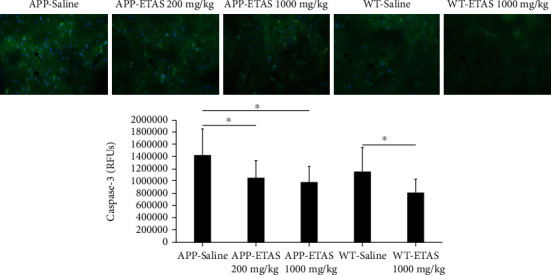
Quantification of caspase-3 in the hippocampus of APP-overexpressed mice treated with ETAS (200 mg/kg and 1000 mg/kg) and WT mice treated with ETAS (1000 mg/kg) for 1 month, respectively. ^∗^*p* < 0.05 APP-ETAS (1000 mg/kg and 200 mg/kg) vs. APP saline-treated mice; ^∗^*p* < 0.05 WT-ETAS (1000 mg/kg) vs. WT saline-treated mice; RFUs: relative fluorescence units. Four mice per group and four sections per mouse were used; green represents capase-3 protein, and blue represents DAPI. Arrows represent fluorescent green staining, indicating positive expression. Magnification is represented ×200.

As described in Materials and Methods, the MWM test was used to monitor cognitive function in APP and WT mice. As depicted in Figure 1, all mice (APP and WT) were trained for 6 days in the MWM and escape latency was recorded at each time point. Results show that the escape latency significantly decreased by 20% (APP saline and WT saline), by 30% (APP-ETAS®50 200 mg/kg), by 38% (APP-ETAS®50 1000 mg/kg), and by 45% (WT-ETAS®50 1000 mg/kg) at day 6 as compared to day 1. The results suggest that mice were well trained in finding the platform. In contrast, we have measured the time stayed in target quadrant when the platform was removed at day 7. Our data indicate that no significant spatial learning was reported between APP saline and WT saline (Figure 1(b)). Escape latency is a very early measure of learning which only involves the time mice take to recognize the platform. Therefore, this might not necessarily be a cognitive factor. However, there was a significant difference in spatial memory between APP saline and WT saline as depicted in Figure 2. Our results show that APP mice administered with ETAS®50 (200 mg/kg, *p* < 0.05) and ETAS®50 (1000 mg/kg; *p* < 0.01) remained in target quadrant for a longer period of time (20% and 30%, respectively) as compared to APP mice receiving saline (Figure 2). To delineate the sources of alterations in cognitive function, e.g., memory in APP mice treated and nontreated with ETAS®50, the hippocampus was stained with specific antibodies (HSP27 and HSP70) as previously described in Materials and Methods. As shown in Figure 3, ETAS®50 administered at solely 1000 mg/kg significantly increased HSP27 and HSP70 in APP mice as compared to APP mice administered with saline. As depicted in Figure 4, our results show that ETAS®50 significantly increased HSP70 (HSPA1A) gene expression (3.14 fold) in comparison with RNA isolated from the right brain slices (inclusive of cortex and hippocampus) of APP+ETAS (1000 mg/kg) treated mice compared to APP saline-treated mice. In addition to HSPs, we have also assessed amyloid *β* in the hippocampus of APP and WT mice treated or nontreated with ETAS®50. It is now well established that amyloid plaques are the main component found in the brains of Alzheimer patients. As such, our results show that *β*-amyloid protein was significantly decreased following ETAS®50 at 200 mg/kg and 1000 mg/kg as compared to APP mice treated with saline (Figure 5). Also relevant, *β*-amyloid protein was significantly higher in APP-saline mice as compared to WT-saline mice. To confirm the effects of *β*-amyloid, an ELISA quantification was performed to assess soluble and insoluble *β*-amyloid. Our data show that both *β*-amyloid (soluble and insoluble) were increased in APP mice as compared to WT mice. ETAS®50 significantly decreased insoluble and soluble *β*-amyloid at both dosages (Figure 6). As such, our data suggest that insoluble *β*-amyloid (13 ng/ml/100 mg tissue) as compared to soluble *β*-amyloid (87 pg/ml/100 mg tissue) might be responsible for the pathological disorders seen in AD [26]. It is well known that plaques and tangles also called tau proteins, emerge in obvious shapes, starting in regions essential to learning and memory and then scatter to other regions of the brain. Interestingly, we have shown that ETAS®50 at both dosages significantly decreased tau protein in APP mice as compared to APP mice treated with saline (Figure 7). Our data also indicate that ETAS®50 reduced caspase-3 in the hippocampus suggesting that ETAS®50 decreased apoptosis in APP-overexpressing mice (Figure 8).

## 4. Discussion

It is well established that the hippocampus is one of the first regions of the brain to be altered in AD, implying confusion and loss of memory, mainly observed in the early stages of the disease [27]. Although treatments may aid to relieve some of the physical or mental symptoms associated with neurodegenerative diseases, there is currently no cure or way to slow the progression of AD.

Recent studies of ETAS®50 have shown that it reduced stress and improved sleep [28]. ETAS®50 has shown significant HSP70 mRNA expression-enhancing activity [28]. Additionally, it has other benefits, including improving heart rate variability, enhancing cognitive performance, and reducing fatigue [29]. Animal models of human diseases that mimic clinical pathology are essential to identify molecular mechanisms along with the development of preclinical trials. As such, first-generation transgenic mouse models that overexpress proteins linked to familial AD (FAD), and mutant amyloid precursor protein (APP) and presenilin have been extensively used to study Alzheimer's disease [21]. Therefore, we have used transgenic mice overexpressing APP in the proposed studies. As previously indicated, the overall goal of our research project was to determine whether ETAS®50 restored memory function through overproduction of heat shock proteins (HSPs) and decreased *β*-amyloid and tau proteins in APP-overexpressed mice. Our results show that APP-overexpressing mice encountered cognitive impairment while treatment with ETAS®50 significantly restored cognitive function using the Morris Water Maze. HSPs attract extensive attention as they play a vital role in preventing protein misfolding and inhibiting aggregation, hence representing a class of proteins potentially involved in AD pathogenesis [30, 31]. It is also well established that HSPs are molecular chaperones that play a protective role against various stressors by preserving protein's homeostasis [32]. Interestingly, our data show that both HSP27 and HSP70 significantly increased in the hippocampus of APP-overexpressing mice after the oral administration of both doses of ETAS®50 over 1 month. These results were confirmed by a significant increase in HSP70 gene expression in the hippocampus following ETAS®50. While the precise pathogenesis of AD is not well understood, accumulation of amyloid *β* (A*β*) derived from the amyloid precursor protein in the brain is believed to induce neuronal dysfunction and death, triggering dementia. Research conducted by Shankar et al. and Prelli et al. [33, 34] suggested that soluble oligomeric forms of A*β* may be contributing to the evolution of AD. A*β* is the main constituent of amyloid plaques or extracellular deposits in the brains of patients with AD [35, 36]. Specifically, the brain of AD patients is marked by increased amyloid *β* (A*β*) deposits, hyperphosphorylated tau aggregates, synaptic losses, and inflammatory responses. APP is the precursor of A*β*, which is a functionally important molecule in its full-length configuration, as well as being the source of numerous fragments with varying effects on neural function. In normal conditions, APP goes to the nonamyloidogenic pathway, which is cleaved by *α*-secretase and produces secreted APP (sAPP)*α*. When APP goes to the amyloidogenic pathway, it will form insoluble A*β* aggregation and produce plaques in the brain, which is the major pathological mark of AD [26]. Thus, decreased production and/or increased clearance of A*β* to reduce the A*β* deposition in the brain may be the promising strategy for preventing cognitive impairment. Asparagus (Asparagus officinalis L.) contains abundant bioactive components, such as flavonoids and carotenoids. Flavonoids may reduce the A*β* gathering and then achieve a neuroprotective effect [37]. For instance, Hesperetin, a predominant flavonoid in lemons and oranges, improves learning and memory impairment by enhancing the antioxidant defense and BDNF signaling [38]. The neuroprotective effects of quercetin are associated with regulating the nuclear factor (erythroid-derived 2)-like 2 (Nrf2), mitogen-activated protein kinase (MAPK) signaling cascades, and PI3K/Akt pathways [39]. Neural stem cells pretreated with lycopene enhanced BDNF level, reduces the oxidative stress and t-BHP-induced cell apoptosis [40]. Further, three carotenoids, cryptocapsin, cryptocapsin-5,6-epoxide, and zeaxanthin, show antiamyloidogenic activity through preventing the fibril formation via disruption of the A*β* aggregates [41]. This evidence supports the potential for foods rich in bioactive ingredients to prevent or reverse age-dependent deterioration. As such, our results indicated that ETAS®50 decreased A*β* accumulation by downregulating the APP expression. Decades ago, a second protein called tau, located inside brain cells, was identified as a possible cause of AD [42]. Of interest, Mietelska-Porowska and Wasik reported that tangles, twisted fibers of tau, form in areas central to learning and memory and then scatter to other regions of the brain [43]. Although in healthy brain areas, tau helps keep the transport system on target, in brain areas where tangles are forming, the twisted strands of tau broadly break up the transport system, preventing essential nutrients to traffic through the cells and resulting in cell death. In agreement with previous studies, our results show that tau protein was significantly expressed in the hippocampus of transgenic mice overexpressing APP. Of interest, the tau antibody used in our study is directed against the phospho-tau pSer199 (one of AT8 epitopes). The serine 199 is a normal site of tau phosphorylation, and this phosphorylation increases in the brain of AD patients. As such, our data shows that ETAS®50 has a direct effect on the standard site of tau phosphorylation as tau was significantly decreased in the presence of ETAS®50 at both dosages. It is well accepted that substantial neuronal death due to apoptosis is a common feature in the brains of patients diagnosed with neurodegenerative diseases. Shimohama reported that apoptotic cell death was observed in neurons and glial cells in patients suffering from Alzheimer's disease [44]. Earlier reports have established that APP and tau undergo caspase-dependent cleavage and that total A*β* and tau production is increased during apoptosis. Also, it has been shown that apoptosis increases levels of A*β*_1-42_ in CHO cells. Interestingly, A*β*_42_ is more prone to aggregate and has been suggested as being the starting point of plaque formation [45]. A*β*_42_ has been reported as being increased in the brain and cerebrospinal fluid after stroke or head trauma [46]. Intriguingly, caspase activation has been shown to occur during cerebral ischemia and head injury, both of which increase the risk for AD by possibly representing either precipitating or triggering events [47, 48]. Caspase activation/apoptosis increases production of A*β*_42_ and tau, consequently triggering and/or exacerbating AD pathology. In a recent study, Hussein et al. reported that L-carnitine modulated epileptic seizures via suppression of apoptosis and autophagy and upregulation of HSP70 [49]. Our data show that ETAS®50 significantly decreased caspase-3, an important marker of apoptosis in the hippocampus.

*Limitations of the study*: future studies warrant the neuroprotective effects of ETAS in adult mice overexpressing APP.

## 5. Conclusions

In conclusion, our findings suggest that ETAS®50 is beneficial for cognitive impairments, reduction of A*β* deposition, and hyperphosphorylation of tau protein in young transgenic mice overexpressing APP. Further, the decrease of caspase-3 expression following the ETAS treatment could facilitate the treatment and prevention of AD. These findings suggest that ETAS®50 treatment can be beneficial in patients with early neurodegenerative diseases by protecting neuronal cells. Future studies warrant the neuroprotective effects of ETAS in adult mice overexpressing APP.

## Figures and Tables

**Table 1:** 

Day	Trial 1	Trial 2	Trial 3	Trial 4
1	SW	NW	SE	NE
2	SW	NW	NE	SE
3	NW	SW	SE	NE
4	NE	NW	SE	SW
5	SE	NW	SW	NE
6	NE	SW	NW	SE
7 (probe)	SW			

## Data Availability

The data supporting the findings of this study are available and will be provided upon request.
